# Septic shock by mechanical ventilation-associated pneumonia

**DOI:** 10.1186/cc12945

**Published:** 2013-11-05

**Authors:** Marcelo Lourencini Puga, Fernanda Guioti Puga, Paulo Eduardo da Rocha Costa, Rodrigo Maia Lima, Edwards Danni Damas da Silveira, Mayra Gonçalves Menegueti, Maria Auxiliadora Martins, Anibal Basile Filho

**Affiliations:** 1 Hospital of Clinicals, Faculty of Medicine of Ribeirão Preto, University of São Paulo, Brazil

## Background

A 69-year-old woman underwent elective surgical repair of an abdominal aortic aneurysm. Intraoperative lesions were intestinal and splenic, requiring performing segmental bowel resection and splenectomy. By hemodynamic instability the patient was maintained on mechanical ventilation in norepinephrine and was transferred to the ICU. After 3 days she had fever, tachycardia, hypotension and anuria, with output fetid and purulent secretion by the tracheal tube. Chest X-ray showed opacity in the right lung; cultures were collected and cefepime initiated empirically for treatment of ventilator-associated pneumonia. *Acinetobacter baumannii *was isolated sensitive only to polymyxin-E in the sample of tracheal secretions. An exchange of antimicrobial therapy was made, but the patient developed refractory shock and died.

## Materials and methods

We report the case of a patient with septic shock.

## Results

Despite the upgrading of intensive therapies with the presence of increasingly prepared professionals and all of the technological and scientific developments that occurred in the last 10 years, sepsis remains a major challenge for contemporary medicine. Mortality rates may vary from 20 to 80%. Several factors contribute to this high mortality rate, such as the growing population of patients aged over 65 years with various chronic diseases, the most frequent use of invasive procedures, increased demand for immunocompromised patients and the development of nosocomial microorganism infections increasingly resistant to antimicrobial agents. Besides the pathophysiology, evidence substantiated that early intervention reduces mortality in severe sepsis and thus several ICUs have sought to improve the quality of clinical management of septic patients. In 2002, the Medical Society of Intensive American (SCCM) and European (ESICM) together with the International Sepsis Forum initiated the Surviving Sepsis Campaign (SSC). The SSC initiative was based on six strategies, including: implement surveillance sepsis; improve the early diagnosis and safety; establish protocols for treatment and early intervention; create programs continuing professional education; proposed therapy post-ICU; and develop global standards for intensive care.

## Conclusions

The emergence of antimicrobial-resistant microorganisms is a growing problem worldwide and this complicates the choice of empirical antimicrobials and can compromise the evolutionary outcome of patients.

